# Tripleurin XIIc: Peptide Folding Dynamics in Aqueous and Hydrophobic Environment Mimic Using Accelerated Molecular Dynamics

**DOI:** 10.3390/molecules24020358

**Published:** 2019-01-19

**Authors:** Chetna Tyagi, Tamás Marik, András Szekeres, Csaba Vágvölgyi, László Kredics, Ferenc Ötvös

**Affiliations:** 1 Department of Microbiology, Faculty of Science and Informatics, University of Szeged, Szeged, Közép fasor 52, H-6726 Szeged, Hungary; mariktamas88@gmail.com (T.M.); szandras@bio.u-szeged.hu (A.S.); mucor1959@gmail.com (C.V.); kredics@bio.u-szeged.hu (L.K.); 2 Doctoral School of Biology, Faculty of Science and Informatics, University of Szeged, Szeged, Közép fasor 52, H-6726 Szeged, Hungary; 3 Institute of Biochemistry, Biological Research Centre, Szeged, Temesvári krt. 62, H-6726 Szeged, Hungary; otvos@brc.hu

**Keywords:** peptaibols, enhanced sampling, molecular dynamics simulations, solvent, principal component analysis, tripleurin

## Abstract

Peptaibols are a special class of fungal peptides with an acetylated *N*-terminus and a C-terminal 1,2-amino alcohol along with non-standard amino acid residues. New peptaibols named tripleurins were recently identified from a strain of the filamentous fungal species *Trichoderma pleuroti*, which is known to cause green mould disease on cultivated oyster mushrooms. To understand the mode of action of these peptaibols, the three-dimensional structure of tripleurin (TPN) XIIc, an 18-mer peptide, was elucidated using an enhanced sampling method, accelerated MD, in water and chloroform solvents. Non-standard residues were parameterized by the Restrained Electrostatic Potential (RESP) charge fitting method. The dihedral distribution indicated towards a right-handed helical formation for TPN XIIc in both solvents. Dihedral angle based principal component analysis revealed a propensity for a slightly bent, helical folded conformation in water solvent, while two distinct conformations were revealed in chloroform: One that folds into highly bent helical structure that resembles a beta-hairpin and another with an almost straight peptide backbone appearing as a rare energy barrier crossing event. The hinge-like movement of the terminals was also observed and is speculated to be functionally relevant. The convergence and efficient sampling is addressed using Cartesian PCA and Kullback-Leibler divergence methods.

## 1. Introduction

Small bioactive peptides like peptaibols have piqued the interests of microbiologists owing to their antibacterial, antifungal, anti-viral, anti-helminth, and anti-tumor properties [1,2,3,4,5], as well as their abilities to elicit plant defense responses [6,7]. Peptaibols are linear, non-ribosomally produced amphipathic polypeptides of fungal origin, mostly comprising a high ratio of unusual amino acid content. Non-standard amino acid residues like aminoisobutyric acid (Aib), d-isovaline (Div), hydroxy-proline (Hyp), and C-terminal alcohol residues like phenylalaninol (Pheol), valinol, etc., along with an acetylated *N*-terminal (Ac) are characteristic for these peptides ranging seven–20 amino acid residues in length. They are synthesized by large modular enzymes called non-ribosomal peptide synthetases (NRPSs), where a single module contains multiple catalytic domains responsible for the incorporation of a single amino acid residue into the peptide chain. Due to the relaxed specificity of NRPSs for residues, the peptaibol groups produced by a fungal strain show considerable heterogeneity [8]. For example, Raap et al. [9] reported that, upon addition of free Aib residues into the medium, the NRPS system of *Emericellopsis salmosynnemata* produces Zervamicin-IIa (containing only Aib residues) as the major secondary metabolite, while upon addition of l/d-Iva, the same NRPS produces Zervamicin-IIb with one d-Iva residue (replacing the first Aib residue in Zervamicin IIa), which indicates the lack of selectivity of NRPS for this substrate. Peptaibols show antibiotic properties by forming pores within biological membranes via aggregation, the implication of which has been widely studied [10,11,12]. Apart from studies on their bioactivity, substantial focus has been given to their three-dimensional structures and folding dynamics [13,14,15,16]. In the protein databank (PDB, www.rcsb.org) about 15 experimentally (X-ray crystallography or NMR) known structures have been documented so far. Alamethicin is the most studied peptaibol so far, closely followed by antiamoebin and zervamicin. Nagao et al. [17] studied the orientation of alamethicin in phospholipid bilayers and found that helical axes of *N* and C-terminals were tilted by 17° and 32° degrees, respectively, to the bilayer normal. The difference in tilt angles for the termini indicates towards a central kink and bends in the alamethicin structure. In fact, such observation has been made for all trans-membrane proteins by Hall et al. [18], who showed that 44% of trans-membrane helices contained a significant kink, proline being the cause 35% of the times. It can be speculated that the occurrence of this bend must have a crucial functional role either during membrane disruption or in the ion-channel formation. 

In this study, we carried out structure elucidation of tripleurins, comprising a newly identified group of peptaibols produced by the fungus *Trichoderma pleuroti*, which causes a green mold disease in the cultivation of oyster mushroom (*Pleurotus ostreatus*) [19]. Tripleurins were also reported as potential growth inhibitors of oyster mushroom mycelia. Not much is known about their behavior and dynamics in different environments and this is the first study to understand their folding behavior in water and chloroform. The choice of chloroform as a solvent was made due to its low dielectric constant (ε_r_ = 4.80) that makes it a suitable mimic of the hydrophobic environment provided by biological membrane systems. It is commonly known that such anti-microbial peptides interact with host lipid bilayers and affect their integrity in several ways. Therefore, we were curious to observe any possible structural changes that might occur in such an environment. LC-MS (Liquid Chromatography-Mass Spectrometry) approaches could not distinguish between isobaric residues, which are therefore marked as Vxx for l-valine or l- and d-isovaline and Lxx for l-leucine or l-isoleucine. Similarly, their 1,2-amino alcohols are referred to as Vxxol and Lxxol. From a mixture of various tripleurins, tripleurin XIIc (referred to as TPN XIIc henceforth) has been selected based on its high yield and lowest number of ambiguous positions. 

We also attempted to understand the evolution of peptide secondary structure based on Ramachandran plots (also referred as phi-psi plots/distribution) through the course of simulations carried out in different solvents. Hollingsworth and Karplus [20] described five main conformation clusters on the plot, namely, α-helices, β-strands, polyproline II (PII) -spirals, γ-turns, and γ′-turns along with an additional ε-region (later named PII’ region) and the bridge region between α-helices and β-strands (δ and δ′). However, only three broad structure types occur in terms of linear groups, i.e., series of residues with the same repeating conformation. These groups are (1) α/3_10_ helices, (2) a group that is largely made of β-strands, and (3) the last group that adopts a PII spiral conformation, ϕ, ψ = (−65, +145), while the left-handed helices may exist as short segments. They also mentioned β-turns, PII-spirals, γ- and γ′-turns, ζα, and ζPII regions as less regular structures. The structural and dynamic study of peptaibols has been quite challenging due to the presence of non-standard residues, their stereochemistry and the lack of experimental structures. The question of simulation time length and adequate sampling remains a bottleneck in most cases involving unfolded structures. To overcome the sampling problem, an all-atom enhanced sampling technique called accelerated molecular dynamics (aMD) has been employed, which provides a non-negative boost to the potential, which reduces the energy barriers between transition states [21,22]. It has been demonstrated that a 500 ns long aMD simulation (using GPUs) could traverse an energy landscape equivalent to a 1 ms long classical MD simulation [23]. Moreover, it does not require any predefined reaction coordinates, unlike other biased free energy calculation methods [24]. We carried out four independent simulations in water and chloroform solvents. The first three simulations were carried out for 500 ns, while the fourth was carried out for 1 μs (1000 ns) using different boost parameters. The dynamics of TPN XIIc was studied in detail using principal component analysis on internal/dihedral angle coordinates and effective sampling was assessed using Cartesian coordinate-based PCA. A relatively new method, named Kullback-Leibler divergence, to measure extent of overlap between probability distribution [25], has been used to discuss adequate sampling and convergence. 

We focused on the folding dynamics of a recently discovered 18-mer peptaibol, Tripleurin (TPN) XIIc, which is an important step to unfold the structural properties of tripleurins and an attempt to correlate their structure and function. It is interesting to analyze the behavior of individual residues of the peptide and their effects on the overall conformation. The primary goal was to obtain a stabilized folded conformation of TPN XIIc, as this knowledge can prove beneficial to design highly bioactive compounds. 

## 2. Results and Discussion

### 2.1. Secondary Structural Populations in Water and Chloroform Solvents 

In this study, we characterized the individual amino acid conformations, along with overall conformation based on the nomenclature described by Hollingsworth and Karplus [20]. The average Φ and Ψ angle values for various conformations on the Ramachandran plot are given in Table 1. A detailed analysis for each residue is necessary for future evaluation of their contribution to the overall structure and bioactivity. For example, Aib has been established as the helix shape promoter during many investigations, and was thus used in many helical peptide-designing studies [26,27,28]. According to Mondal et al. [29], the addition of an extra α-methyl group limits the range of allowed φ-ψ (phi-psi) torsion angles of Aib in comparison with alanine and restricts the energetically accessible conformational space. It is clear that Aib constraints the backbone by reducing the degree of freedom of movement of the peptide. Another important aspect to be considered here is the achirality of Aib, which enables it to readily form right or left-handed helices depending on the screw-sense of preceding or following residues in the peptide chain. It has also been established that the presence of Aib renders the resulting peptide protease-resistant. Wada et al. [30] synthesized an amphipathic helix peptide using Aib, which showed potent resistance to trypsin and pronase. Additionally, d-residues in general have been shown in various cases to enhance the stability, activity [31], and resistance to proteolytic enzymes [32,33]. It is therefore beneficial to observe the effects of these residues exerted on the structure and function of peptides, and whether this effect is dependent on their number in the peptide chain. 

**Figure 1:**
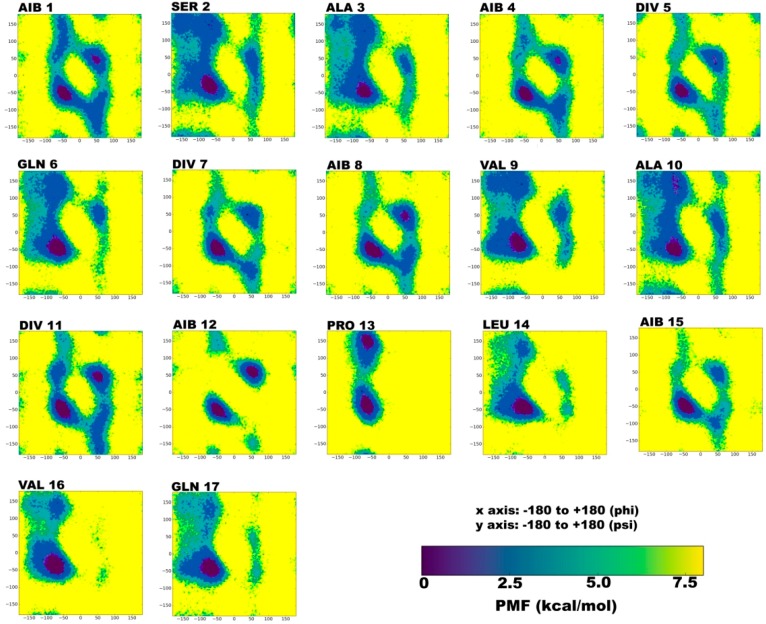
Reweighted PMF (potential of mean force) phi-psi dihedral angle plots for each TPN XIIc residue during explicit water aMD simulation. The x and y axes range from -180 to +180. The darkest violet regions indicate toward minimum energy secondary structural regions favored by each residue during the simulation.

**Figure 2:**
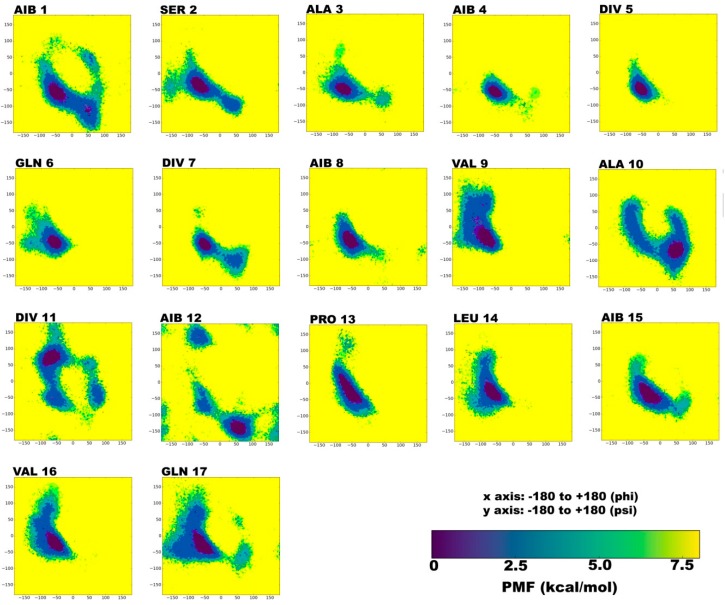
Reweighted PMF (potential of mean force) phi-psi dihedral angle plots for each TPN XIIc residue during explicit chloroform aMD simulation. The x and y axes range from -180 to +180. The darkest violet regions indicate toward minimum energy secondary structural regions favored by each residue during the simulation.

**Figure 3:**
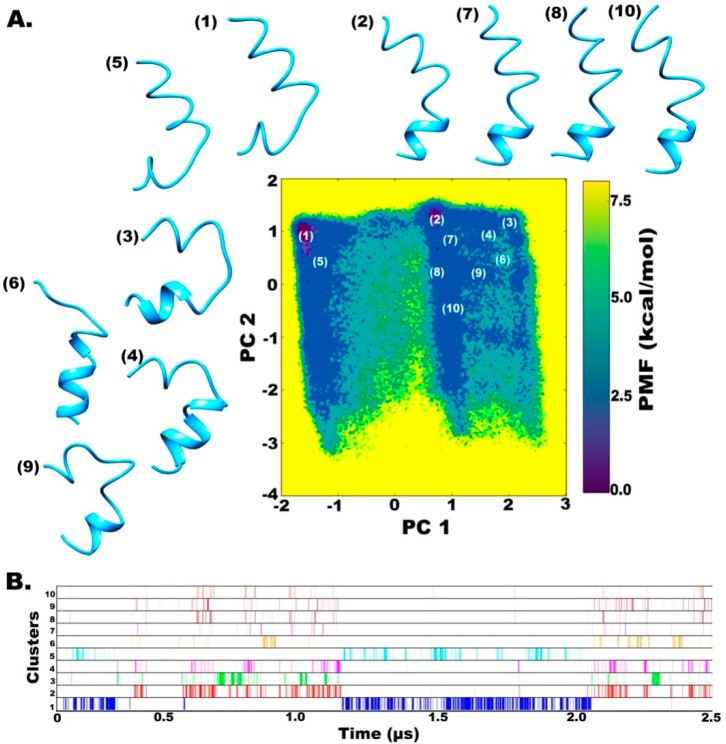
(**A**) Reweighted free energy landscape of the first two principal components calculated from dihedral angles, phi-psi, for better clustering based on internal motions. (**B**) Diagrammatic representation of cluster distribution along the simulation trajectory in water.

**Figure 4:**
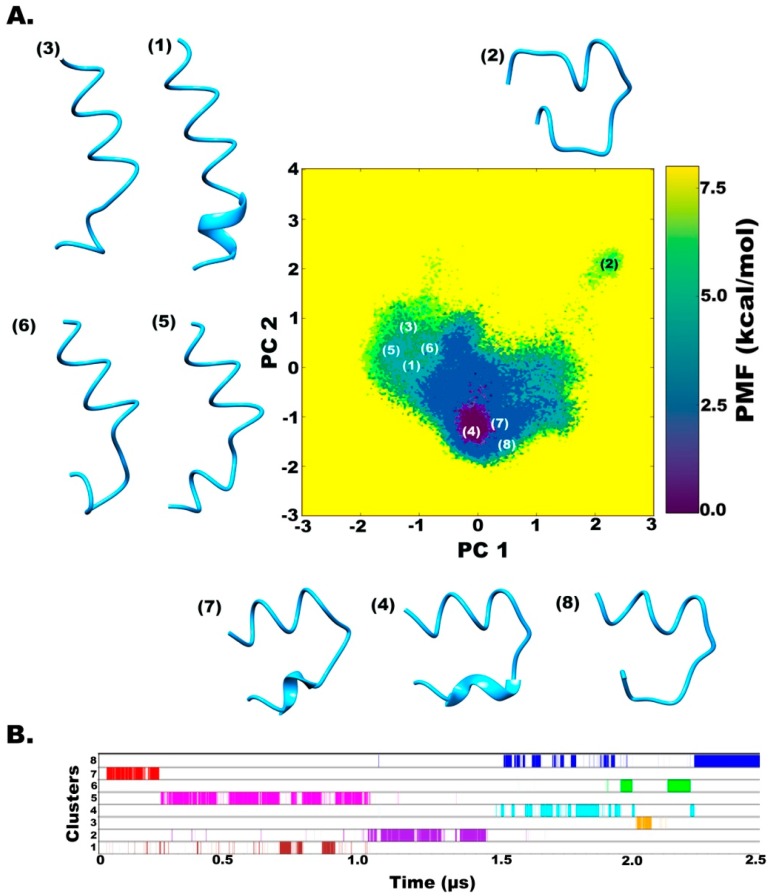
(**A**) Reweighted free energy landscape of the first two principal components calculated from dihedral angles, phi-psi, for better clustering based on internal motions. (**B**) Diagrammatic representation of cluster distribution along the simulation trajectory in chloroform.

The reweighted phi-psi plots have been constructed for each individual residue of TPN XIIc. The color scale in Figure 1, Figure 2, Figure 3 and Figure 4 denotes potential of mean force or PMF (kcal mol^−1^) to characterize energetically favored conformations that were visited during simulations. The darkest violet regions denote the lowest energy minimum. In water solvent, the energy minima for non-standard residues, Aib1, Aib4, Div5, Aib8, Div11, and Aib12 lie in both right and left-handed α-regions. This fluctuation is attributed to the achiral nature of Aib residue and the propensity of d-residues towards left-handed conformations. It has also been noted that chirality of isovaline (Iva) does not highly impact the screw sense of whole helical structure, possibly because of the limited difference in the length of its two side chains [34]. The Div residues are found in right-handed helical peptides, where the screw sense is governed by the L-isomeric amino acids present in their sequences. Another study reported that Iva-rich peptides favor beta-bends and 3_10_ helices [35]. A study by Shenkarev et al. [36] compared structural and functional characteristics of Antiamoebin I (Aam-I) with Zervamicin IIb (Zrv-IIb). Both molecules acquire very similar structural topologies in membrane-mimicking environments, although they significantly differ at the *N*-terminal (1–8 res.) due to high presence of Aib, Div, and Gly achiral residues in Aam-I. On the other hand, Aam-I and ZRV-IIb show markedly different dynamic properties owing to the former’s high ‘motional’ propensity, which may be caused by conformational exchange (characterized by positive and negative ϕ/ψ torsion angles) undergone by Aib, Div, and Gly residues. They hypothesized that such conformational exchange may be responsible for its high solubility in water solvent, which may prevent Aam-I to effectively perturb lipid bilayers. It is experimentally proven that Aam-I shows lower binding affinity to lipid vesicles than Zrv-IIb, and therefore, also shows weaker bioactivity. This implies that although the presence of achiral residues like Aib is necessary for helix formation, the high fluctuation induced by them may render the peptide less bioactive. In case of TPN XIIc it is clear that the right-handed helical sense is energetically more favorable than the left-handed, although both right and left-handed helical states are visited in the case of Aib and Div residues. Due to such observations, it is difficult to believe that highly diverse peptaibol sequences are driven by evolution. The high heterogeneity within a single group of peptaibols indicates the lack of selectivity in NRPS proteins. On the other hand, the knowledge of correlation between the presence of certain residues and their functional relevance may help in the design and synthesis of relevant biomolecules that could be exploited as therapeutics and biocontrol agents.

The energy minima for almost all standard residues like Ser2, Ala3, Gln6, Leu14, Val16, and Gln17 lie in the right-handed α-region, while the other secondary structural states could only be reached at a difference of 2 kcal mol^−1^. One such state is the bridge between α- and β-regions, known as the δ region which represents β-turn formation in the peptide chain and continuous formation of β-turns gives rise to β-bend ribbon spirals. Most standard residues of TPN XIIc show considerable population in this region even if at a higher energy scale (~4 kcal mol^−1^). On the other hand, Pro13 traverses poly-proline II region, which is expected according to Brown et al. [37] as proline (Pro) shows the highest PII propensity. 

In water environment, TPN XIIc peptide shows fluctuating propensity for i + 3 → i and i + 4 → i type H-bonds (Table 2). Gln6 → Ala3 (19%), Val9 → Gln6 (15%), Ala10 → Div7 (11%), Aib12 → Val9 (4%), and Val16 → Pro13 (19%) are examples of i+3 → i type H-bonds, while Div7 → Ala3 (14%), Aib12 → Aib8 (12%), Ala10 → Gln6 (14%), Div11 → Div7 (14%), Gln17 → Pro13 (21%) and Pheol18 → Leu14 (44%) are examples of i+4 → i type H-bonds. On the Ramachandran plot, α- and 3_10_ regions are present almost in the same subspace and are easily inter-convertible in terms of energy difference. Based on this analysis it can be deduced that a loose spiral-like conformation is favored instead of a strict helical conformation (except at C-terminal). 

On the contrary, reweighted phi-psi plots obtained from chloroform simulation indicate towards a considerably different folding pattern (Figure 2). The energy minima for Aib1, Aib4, Div5, Div7, Aib8, and Aib15 lie only in the right-handed α-region, unlike in the water solvent, where both right and left-handed states are visited. A rather unusual behavior is exhibited by Ala10, which favors the classic gamma turn formation, while the consecutive Div11 residue favors inverse gamma turn region. This would probably bend the backbone in a slight ‘S’-shape or an outward kink. 

The corresponding H-bond Aib12 → Ala10, which occurs in 44% of the simulation time, also indicates towards a strong backbone reversal at this position due to formation of a gamma turn. The H-bonds formed during this simulation have been summarized in Table 3. This observation can be attributed to the non-polar nature of chloroform environment. A recent study on the behavior of alanine dipeptide in explicit chloroform and water solvents by Rubio-Martinez et al. [38] described that C_7_^eq^ conformation only appears as a low energy minimum in chloroform. Standard residues like Ser2, Ala3, Gln6, Val9, Leu14, Val16, and Gln17 show energy minima strictly in the right-handed α-region. 

In conclusion, the TPN XIIc peptide shows higher propensity for spiral-like helix at the *N*-terminal and α/3_10_ helix at the C-terminal with a slight backbone bend in water solvent, and for γ-turn in the central region that may induce backbone reversal in chloroform solvent. The C-terminal mostly folds into a 3_10_ helix in both solvents, but is disrupted by few gamma turn-inducing bonds like Val16 → Leu14 (17% occurrence) in the chloroform solvent. 

### 2.2. Clustering based on Free Energy Landscapes (FEL): Vision through Principal Component Analysis

In addition to the native structure, we were also interested in the folding properties of TPN XIIc under the effect of different solvents. Peptide folding is a dynamic process of evolution of intermediate ensemble states in a back-and-forth fashion, which should eventually result in a folded state. The results of the simulations were analyzed by principal component analysis (PCA) to reduce the dimensionality of data and to visualize the free-energy landscapes, resulting in the intermediate states and their path to achieve the final folded state [39]. A Cartesian coordinate PCA (cPCA) based on the overall motions of a peptide can distort this idea and present ambiguity in the spread of free-energy basins [40], therefore dihedral-angle based PCA (dPCA) [41] was employed to only include internal motions (defined by ϕ, ψ dihedral angles) for the peptide folding process. A free energy landscape based on internal motions (projected along first two principal components PC1 and PC2 using μ(q_1_, q_2_) = −k_B_T lnP(q_1_, q_2_) provides accurate results of the minimum energy wells and barriers between them, especially for systems undergoing large changes [42]. All dihedral PCA-based FEL plots have been reweighted for calculation of PMF and accurate description of the energy minimum. 

The two simulations were carried out for 500 ns × 3 using different starting structures along with another 1000 ns long simulation. All simulations have been combined (2.5 μs) for the clustering procedure. The clustering procedure involves identification of isolated peaks in a three- or five-dimensional density map obtained from the trajectory’s principal component distribution. Each point on this plot signifies all the structures that have PC values closest to that point. All peaks with density higher than a given threshold are selected, which correspond to a distinct cluster. The darkest violet regions on the FEL map (Figure 3A) show the lowest energy conformation clusters, which denote the metastable states of this peptide. As can be observed on the reweighted maps, at least two different regions of energy minima can be identified in the case of water solvent, while only one prominent energy minimum is revealed in chloroform. 

The full trajectory was clustered into 10 major representative groups (Figure 3A). The two prominent clusters, (1 and 2 based on 12% and 5% occurrence during the simulation, respectively) on the FEL map are separated by an energy barrier of at least 5 kcal mol^−1^. The representative structures show major differences in the C-terminal folding, which is a loose spiral in clusters 1 and 5, but a highly folded helix in cluster 2. A similar observation was made by calculating PMF (in kcal mol^−1^) for end-to-end distance of the peptide over whole trajectory where the distance from ~18 to 21 Å denotes a large energy minimum basin which means that a slightly bent peptide backbone is energetically favored (Supplementary file 1, Figure S1). Structures 7, 8, and 10 are closer to cluster 2 and represent a slightly bent helical folded structure unlike clusters 3, 4, 6, and 9, which are highly curved folded structures. Figure 3B shows a diagrammatic distribution of the inter-conversion between these clusters. The two main clusters denoting two deepest energy minima are revisited several times during the course of simulation but show an almost mutually exclusive occurrence with respect to each other. Cluster 5 occurs intermittently with cluster 1 while the rest of them show inter-conversion with Cluster 2. This suggests that the formation of an alpha-helical C-terminal fold is an uncommon energy barrier-crossing event and would not have been effectively sampled using short-timescale classical MD approaches. Another observation is the similarity of structures sampled in all independent simulations (clustered in the same group based on dihedral PCA), which signifies satisfactory sampling.

In the case of chloroform solvent (Figure 4A), eight different clusters were obtained. Interestingly, the major conformations obtained through the first three independent 500 ns long simulations showed a backbone curvature in the folded peptide that is observed as the region of the deepest minimum denoted by Cluster 4 (13% occurrence throughout the combined trajectory). This is expected due to the presence of γ-turns that cause backbone reversal. It was determined that the Aib12 → Ala10 bond results in an energetically stable backbone reversal by i + 1^th^ residue Div11 which populates the γ’-region (inverse gamma turn or C_7_^eq^ conformation) on the phi-psi distribution plot. An end-to-end distance of 14 Å (indicating backbone reversal) lies in an energy minimum, while the unfolded conformation lies in another minimum (Supplementary File 1, Figure S1). The last 1 μs long simulation mainly resulted in a distinct linear backbone conformation represented by Cluster 1 (11% occurrence throughout the combined trajectory) that was observed for an insignificant period during previous simulations. The energy barrier between the two states (Cluster 4 → Cluster 1) is ~4 kcal mol^−1^, i.e., the conversion from a highly bent to a linear conformation is a rare event and requires a longer continuous simulation to be achieved with aggressive dihedral boost parameters. It is also supported by the end-to-end distance value of 27 Å (linear backbone) which is achieved after crossing a barrier of 5 kcal mol^−1^ (Supplementary File 1, Figure S1). There is a 6 kcal mol^−1^ energy barrier between the unfolded conformation represented by cluster 2 and folded conformation of cluster 4, which was achieved immediately after 200 ns. Clusters 4, 7, and 8, on the other hand, show interconversion quite a few times between 700 to 900 ns with respect to each other (Figure 4B). 

It should be carefully considered that the energetically stable state of TPN XIIc in chloroform is a highly bent shape that is not ideal for membrane-spanning peptides. The ideal linear conformation was only obtained after crossing the energy barrier that remains stable afterwards.

### 2.3. Understanding the Dynamics of TPN XIIc through Cartesian PCA

**Figure 5:**
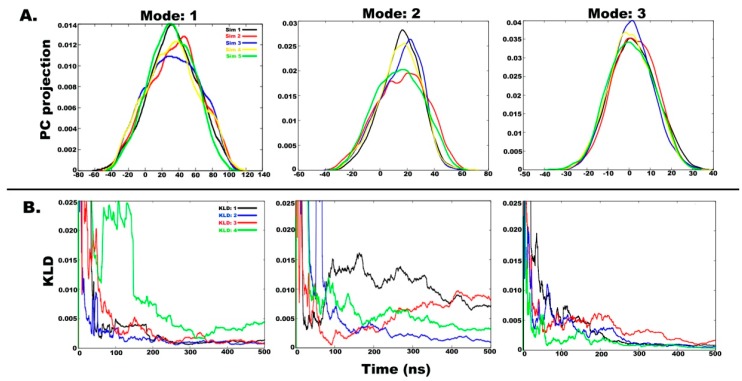
(**A**) Histograms of projection of principal components 1, 2, and 3 for all four simulations in water, where the last 1 µs long simulation is treated in 2 parts. Histograms were calculated using a Gaussian kernel density estimator. (**B**) A measure of overlap between histograms from independent simulations calculated using the Kullback-Leibler divergence method. The slope values lying below 0.025 indicate convergence between two independent runs.

In the previous section, we presented peptide dynamics based on free energy landscapes obtained through dihedral PCA, which only accounts for the internal motions. In this section, we shall uncover the overall motions of the peptide to attain various metastable states. The combined trajectory (2.5 μs) was divided into five parts of equal time length, i.e., ~500 ns. This means that each individual trajectory and the last 1 μs long simulation divided in two equal parts were considered. Figure 5A shows the histograms for projection of coordinates along the first three eigenvectors (i.e., the first three modes with highest eigenvalues). The trajectories are named as “Sim1” to “Sim5” and the extent of PC overlap signifies convergence between independent runs. The degree of overlap suggests that the independent simulations sampled similar conformational space [36]. The essential PCs 1, 2, and 3 obtained from five separate trajectories accounting for 24%, 15%, and 11% of overall motions, respectively, show considerable overlap. A considerable overlap between first PC histograms (Mode 1) shows very similar motions of the peptide during the first and last simulations while the second PC histograms (Mode 2) show major similarity between the second and last simulations. The third PC histogram (Mode 3) is similar for all five independent trajectories. 

The quantification of convergence and statistical significance in macromolecular MD simulations often presents a challenging task, and more so, in case of peptide folding calculations. A quantitative measure of extent of overlap between any probability distribution is the Kullback-Leibler divergence (KLD) method that can indicate satisfactory sampling [25]. By measuring the overlap of PC histograms as a function of simulation time, we can assess the convergence of dynamic properties of simulations. The principal components give an idea of conformational states that have been visited during the simulation. Two independent simulations started from different configurations should eventually begin to sample the same conformations [43]. The extent of overlap between such simulations will indicate that satisfactory sampling has been attained. The KLD method can be applied to measure the extent of overlap of any quantity (e.g. RMSD), but we have chosen PC histograms as they provide a clear idea of the dynamics of the peptide. A rapidly decreasing slope of KLD as a function of time indicates convergence between two independent simulations. As per previous studies, we have selected the KLD value of 0.025 as the cutoff for convergence. When the KLD slope hits below 0.025, the two simulations will have converged. 

Figure 5B shows KLD as a function of time between subsequent histograms from five different simulations for PCs 1, 2, and 3 (accounting for 50% of total motion). “KLD:1” denotes divergence between Sim1 and Sim2, “KLD:2” between Sim2 and Sim3, “KLD:3” between Sim3 & Sim4 and “KLD:4” between Sim4 and Sim5. It is evident that the slope of KLD:1, KLD:2, and KLD:3 values for PC 1 does not change significantly after 200 ns, therefore, signifying convergence. KLD:4, on the other hand, shows a drop to 0.025 at around 300 ns before rising again at 350 ns which means that a wider conformational space was sampled during 1 μs long simulation. The KLD values for PC 2 are highly divergent and show that convergence was not obtained for this mode of motion while KLD for PC 3 shows high convergence. This analysis also indicates that the minimum sampling time required for TPN XIIc peptide in water is 500 ns using accelerated MD. It can be safely stated that all major metastable states of TPN XIIc in aqueous medium have been sampled during independent simulations. 

**Figure 6:**
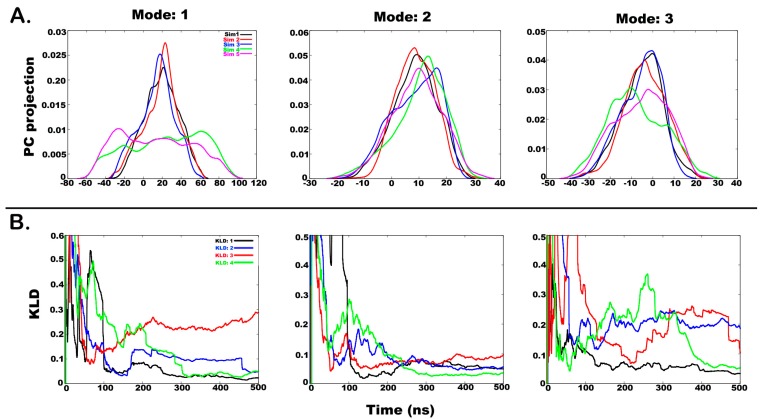
(**A**) Histograms of projection of principal component 1, 2, and 3 from all four simulations in chloroform where the last 1 µs long simulation is treated in 2 parts. Histograms were calculated using a Gaussian kernel density estimator. (**B**) A measure of overlap between histograms from independent simulations calculated using Kullback-Leibler divergence method. The slope values lying below 0.025 indicate convergence between two independent runs.

A similar analysis for chloroform solvent divulged some interesting results. Figure 6A shows the histograms for projection of coordinates along the first three eigenvectors (i.e., the first three modes with highest eigenvalues). The overlap of first PC histograms (mode 1 accounting for 53% of overall motions) shows similarity between the first three independent simulations, while the fourth 1 μs long simulation shows a drastically different mode of motion. However, such a stark difference is not observed for the second PC (mode 2). 

Figure 6B shows KLD as a function of time between subsequent histograms from five different simulations for PCs 1, 2, and 3 (accounting for 68% of total motion). It can be noted that the slope of KLD:1 and KLD:4 values for PC 1 does not change significantly after 300 ns, therefore, signifying convergence between Sim1 and Sim2 and Sim4 and Sim5, respectively. KLD:2 values for PC1 reach a value of 0.05 at around 150 ns before increasing again. This means that Sim2 and Sim3 initially sampled similar structures before diverging on separate conformational paths. KLD:3 values between Sim3 and Sim4 remain divergent, which is expected due to very different conformational spaces covered during the previous 500 ns long simulations and the latest 1 μs long simulation. As mentioned before, when KLD slope rises beyond 0.025, it indicates that two independent simulations diverged in the conformational space. The occurrence of a linear backbone structure in the last 1 μs simulation in contrast to a highly bent structure in previous simulations can be believed to be the reason for this difference. As the peptide undergoes significant motion around the Div11-Aib12-Pro13 hinge region to attain a straight backbone, a new metastable state is achieved, which is comparable to structures obtained from water simulation. Therefore, a combination of all simulations has resulted in a nearly complete conformational landscape for the peptide of interest in chloroform solvent. KLD values for PC2 show high convergence, while for PC3, the high KLD:4 values for the two parts of the 1 μs long simulation shows that highly divergent internal motions have occurred in the peptide. 

In some instances, the KLD value rises again after hitting the cutoff once, which indicates that the two simulations diverged and sampled different conformational spaces as the simulation progressed. In the case of chloroform simulation, the last 1 µs long simulation resulted in a state not observed in previous simulations. This is a high energy metastable state that could only be observed due to the use of aggressive boost parameters during accelerated MD. That means that the first three simulations did not sample the entire conformational space (shown by increasing slope of corresponding KLD: 3) and hence could not be considered converged. This puts a question mark on the use of KLD as a method of assessing convergence as if the fourth simulation had not been carried out, the previous three simulations would indicate perfect convergence and that is clearly not true. Our rationale lies in the fact that chloroform is a very restrictive solvent for peptide folding which is clear from our results. The conformational landscape covered in the fourth simulation is a higher energy state (Supplementary Figure S1, linear peptide) that could not have been achieved during shorter simulations. This shows that 500 ns long runs are not adequate to attain the higher energy state in chloroform. The peptide seems to be stuck in a single energy state in chloroform, while it is highly dynamic in water and jumps through various metastable states with relative ease. Irrespective of the starting configuration, all simulations must, at some point, begin to sample the same space which could not be observed in the case of chloroform. However, the combined trajectory of 2.5 µs surely indicates adequate sampling. This is a drawback of using such a statistical method for addressing convergence, especially when enhanced sampling methods have been used in solvents other than water. Nevertheless, it is more reliable than previous methods of considering convergence criteria as RMSD. 

Along with the discussion on convergence of PC space that conveys the dynamics of peptide folding, it was interesting to consider structural convergence as well [44]. Based on Good-Turing formalism [45,46,47] applied on the root-mean-square-deviation values it could be deduced, that slightly higher structural convergence is achieved in chloroform than in water (Supplementary file 1, Figure S2). This indicates that the folding of the TPN XIIc peptide is very dynamic in an aqueous environment and can probably result in many unfolded or intermediate states, even though all energetically stable states have already been achieved. A biological membrane mimicking environment, like chloroform, has a stabilizing effect on peptide dynamics and requires longer time scales to be able to sample higher energy states. A similar study by Levy et al. [48], reported differences in the folding pattern of Ala_12_ peptide in vacuum and water. The peptide was able to achieve its native state in vacuum, while it was destabilized in water and achieved a β-state as native. Additionally, a comparison between different folding and unfolding time scales for Ala_12_ in hydrophilic and hydrophobic solvents was attributed to the higher entropy of native state in water. 

For most proteins, their interactions with organic solvents depend on hydrophobic core destabilization, side-chain exposure, and favoring of hydrophilic protein surface. The flexibility of a peptide and its associated entropy are products of various intermolecular interactions with the solvent. For example, the interaction of water molecules with the peptide polar groups can lead to the destabilization of a strict helical structure and render it in coil-like formation to make the polar groups more accessible. Clearly, any peptide, including TPN XIIc shows higher flexibility in water than its organic counterpart, which was also evident by comparing the extent of conformational landscape covered. A similar observation was reported by Levy et al. [48], who explained that random coils are more prevalent in water as the loss of intra-molecular interaction is compensated by intermolecular interactions with water molecules. On the contrary, while they reported two distinct energy wells for Ala_12_ falling in α-helical and β-sheet regions, we did not observe such distinctive characterization in water or chloroform for TPN XIIc. This indicates that TPN XIIc possesses a stronger propensity for the helical conformation, and due to its amphipathic nature, it can access similar secondary structural states in hydrophilic and hydrophobic environments. The largest difference observed upon change of solvent is the overall bent in the peptide linearity. The peptide seems to undertake its biggest motion around the Div11-Aib12-Pro13 region, rendering either a hairpin like bend or a linear shape. In relation to that, it was noted that the highly bent, hairpin-like shape observed mainly in chloroform occurred due to burying of hydrophilic residues like Gln6 and Gln17 inside the curve, while the hydrophobic residues like Val9, Ala10, Pro13, Leu14, and Val16 are outside, facing the hydrophobic solvent. The same hydrophobic residues shall form the interior of an ion-channel with other peptide units. Thus, it can be stated that due to the effect of a hydrophobic solvent, all hydrophobic residues are aligned to interact with their surroundings. The two energetically stable states obtained in water are relatively linear, where the masking of hydrophobic residues is not possible, but inter-molecular interaction between water and polar residue Gln17 in Cluster 1 may explain the loss of helical structure and its replacement with a loose coil. Probably that is the reason of higher entropy and flexibility in the case of the TPN XIIc peptide. A detailed study of peptide folding characteristics and the corresponding ensemble identification with the help of enhanced molecular dynamics methods has been proven crucial in elucidating the mode of action of their bioactivities. Characterization of bioactive peptides is necessary to identify structural features that are crucial for bioactivity and utilize this information to synthetically design novel therapeutics.

## 3. Materials and Methods 

### 3.1. Sequence Selection

A recent study identified and published a new group of 18-residue peptaibols produced by *T. pleuroti*, named as tripleurins [19]. Out of 24 reported tripleurin sequences, three compounds (Tripleurins VI, VIIIb, and XIIc) were present in the highest area percentage with 7.9%, 10.0%, and 12.0% on the mass spectrometric chromatogram extracted at *m*/*z* of sodiated molecular ions [M + Na]^+^, respectively. Tripleurin XIIc, an 18-residue long sequence with five ambiguous Val/Div and one Leu/Ile position, was selected. Its primary structure is: 

AcAib^1^-Ser^2^-Ala^3^-Aib^4^-Vxx^5^-Gln^6^-Vxx^7^-Aib^8^-Vxx^9^-Ala^10^-Vxx^11^-Aib^12^-Pro^13^-Lxx^14^-Aib^15^-Vxx^16^-Gln^17^-Pheol^18^

The ambiguous residue positions were predicted based on the sequence of non-ribosomal peptide synthetase (NRPS) proteins using the antiSMASH database server [49]. Positions Vxx5, Vxx7, and Vxx11 were predicted to be D-isovalines, Vxx9 and Vxx16 were predicted to be valines, while Lxx14 was predicted as leucine. This prediction adds a high number of d-amino acid residues, which may have a strong influence on the overall structure and bioactivity. 

### 3.2. Partial charge Calculation and Force Field Library Generation for Non-Standard Residues

**Figure 7:**
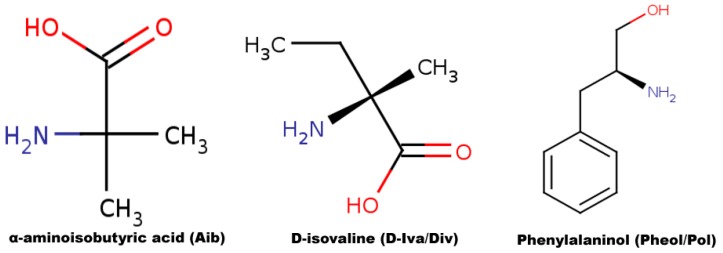
Graphical representation of 2D molecular structures of Aib, Div and Pheol/Pol.

As famously known, fungal peptaibols are characterized by their unusual amino acid content. In the selected sequence, Aib, Div, and Pheol are non-standard residues. A graphical representation of their 2D structures is provided in Figure 7. Both Aib and Div are derivatives of the alanine, in which one methyl group is present as the side-chain. An additional methyl group (-CH_3_) is attached to the Cα carbon atom as the side-chain in Aib, while an ethyl group (-CH_2_CH_3_) is attached in the d-Iva residue. The R.E.D server was used for calculation of their partial charges and creating force field libraries [50]. R.E.D stands for RESP ESP charge derive [51]. RESP (restrained electrostatic potential) was used to calculate the charges with a HF/6-31G(d) basis set and Gaussian09 [52] as quantum mechanical program interface. The charges for Aib and Div were calculated along with a few other standard amino acids. The charges calculated for standard residues were used to confirm with existing “leap” libraries. For each residue, two conformations, i.e., alpha helix (Φ = −63.8, Ψ = −38.3) and beta sheet or C_5_ (Φ = −157.2, Ψ = 161.9) were used. These were modified and generated using Avogadro [53] based on the strategy described by Cieplak [54]. The terminal residue phenylalaninol (Pheol) was also parameterized using a slightly different strategy, where two molecules, ethyl alcohol and phenylalanine were used to form the Pheol unit. The results include calculated charges and a script to make force field libraries for these forces. The TPN XIIc sequence was built by supplying residue units from scratch using “tleap”. The parameters for each non-standard residue are provided in Supplementary file 1. 

### 3.3. Accelerated Molecular Dynamics Simulations of TPN XIIc 

The unfolded peptide conformation prepared by using the ‘tleap’ module of AmberTools18 [55] was solvated in water and chloroform solvents. For water solvent, TIP3P water model was loaded from the AmberTools18 solvents library and 2657 water molecules were added with a box size of 44.34 × 59.11 × 42.85 Å and a volume of 112332.666 Å^3^. The Amber solvent library was also loaded for chloroform, which added 1021 CHCl_3_ molecules with a box size of 49.608 × 59.514 × 67.986 Å and a volume of 200719.862 Å^3^ to the system. The Amberff14SB force field was used to prepare and minimize all systems. 

As classical molecular dynamics (cMD) offers limited utility in terms of shorter time scales, a relatively new approach named accelerated molecular dynamics (aMD) was adopted for this study to enhance sampling. It is a bias potential function introduced by Hamelberg et al. [21,22], which can be used to make the simulation “jump over” high energy barriers and to sample rare events. A detailed discussion is provided elsewhere [56].

All systems were prepared for aMD in six consecutive steps, i.e., (a) minimization (conjugate gradient followed by steepest descent method) of solvent for 20,000 cycles while keeping peptide under restraint, (b) water movement at 300 K under isothermal and isobaric (NTP) conditions while keeping peptide under restraint, (c) minimization of the whole system for 20,000 cycles, (d) heating from 0 K to 300 K under isothermal & isovolumetric (NVT) conditions while keeping peptide under restraints, (e) relax the system at 300 K for 0.5 ns while keeping peptide heavy atoms under restraint, and (f) relax system at 300 K under NTP conditions for 5 ns. The temperature scaling was carried out using Langevin thermostat while the pressure was regulated using the default Berendsen barostat for all corresponding calculations. SHAKE bond length constraints were applied on all bonds involving hydrogen. A short classical MD run to obtain average dihedral and potential energies (in kcal mol^−1^) was also carried out for 100 ns at 300 K temperature and periodic boundary condition was used with constant pressure using Berendsen barostat in each case.

The two systems, unfolded peptide in water and chloroform, were simulated using aMD for 500 ns with three independent starting structures (500 ns × 3) along with a 1000 ns long simulation making a combined simulation time of 2.5 µs. All simulations were carried out at 300 K temperature, 2 fs time step, and energies and boost information was written down at every 1000 steps. The electrostatic interactions were calculated using PME (particle mesh Ewald summation) [57] and long-range interactions were also calculated with a cutoff of 10.0. The temperature scaling was carried out using Langevin thermostat without pressure scaling during aMD. The SHAKE algorithm was applied on all bonds involving hydrogen. The GPU machines available through the NIIF clusters of Hungary were utilized for all aMD simulations. All simulations were carried out using the *pmemd.cuda* implementation of Amber14, which is also available at the cluster. 

AMD can be carried out using three criteria (i) independently boosting the torsional terms of the potential (iamd = 2) or (ii) the whole potential at once (iamd = 1), and (iii) to boost the whole potential with an extra boost to torsions (iamd = 3). The third criterion seemed to be an appropriate choice, as dihedral-only aMD boost is known to enhance the convergence of the underlying free energy landscape by 5-fold in comparison to classical MD, but the dual boost option provides a better reweighting distribution [56].

The extra parameters E_dihed_, α_dihed_, E_total_, and α_total_ were calculated, as required in Equation (1):

E_dihed_ = V_avg_dihed_ + a_1_ × N_res_, α_dihed_ = a_2_ × N_res_/5 E_total_ = V_avg_total_ + b_1_ × N_atoms_, α_total_ = b_2_ × N_atoms_ (1)

where N_res_ is the number of peptide residues (19, with an addition of Ace at the *N*-terminal), N_atoms_ is the total number of atoms in the system which were 8243 and 7462 in the case of water and chloroform solvent systems, respectively. V_avg_dihed_ and V_avg_total_ are the average dihedral and total potential energies obtained from the 100 ns long cMD run in each solvent. The various parameters used for all aMD simulations have been summarized in Table 4. 

In the water simulation, application of smaller boost coefficients b1, b2 = 0.16 results in very small boost (~5 kcal mol^−1^) and the folding event could not be observed for 500 ns. Therefore, comparably aggressive boost parameters, i.e., b1, b2 = 0.30 and b1, b2 = 0.20 were applied to speed up the folding process. It shall also be noted that the total potential energies obtained for the chloroform system was much higher (~−7335 kcal mol^−1^) than in water systems (~−25000 kcal mol^−1^). The chloroform solvent-based simulation showed unexpected behavior when the boost was applied to the potential energy (iamd = 3 or iamd = 1). Even a small potential boost defined by coefficient values of b1, b2 = 0.01 and 0.08 (according to Equation (1)) resulted in extremely high energy values and the calculation could not proceed. Similar results were obtained when a bigger system comprised of more chloroform molecules was tested. It was suspected that the chloroform system already possesses a very high potential energy and even a small boost to the potential increase in the system energy to enormous levels. Subsequently, it was decided to apply only torsional boost (iamd = 2) to this system. To the best of our knowledge, this is the first study on the application of accelerated MD on a peptide solvated in the chloroform solvent. Different values of coefficients a1, a2 and b1, b2 were tested and applied, as summarized in Table 5.

### 3.4. Tools for Simulation Analysis

The calculation of dihedral angle populations and hydrogen bond calculations were carried out using the *cpptraj* [58] module. The dihedral based PCA and Cartesian coordinate PCA were also done using the *cpptraj* module. For dihedral PCA, the phi psi torsion angles are calculated for all residues and the covariance matrix is calculated. The eigenvectors were calculated based on the covariance matrix. The first two principal components are reweighted by the Maclaurin series expansion method. The Cartesian PCA was also carried out with RMS-fit (root-mean-square) to first frame to remove global translation/rotation followed by RMS-fit to average structure calculated in the previous step. The covariance matrix was calculated and diagonalized. The common eigenvectors were calculated and the individual trajectories obtained from water and chloroform were projected on PCA space. The principal component (PC) histograms were obtained for each of the four simulations (replicas) carried out in both solvents. As the last 1 µs long simulation is being treated in two parts, we considered PC histograms from five distributions, i.e., Sim1 to Sim5. The extent of overlap between such replicas in the same solvent should provide an idea of convergence. The Kullback–Leibler divergence (KLD) [25] between the principal component histograms from two replicas (e.g., between Sim1 and Sim2, Sim2 and Sim3 and so on in each solvent) over time was calculated. The time-dependent KLD is calculated as:

(2) $$\mathrm{KLD}(t)=\sum_{i}P\left(t,i\right)ln\left(\frac{P\left(t,i\right)}{Q\left(t,i\right)}\right)$$

where *P*(*t*, *i*) and *Q*(*t*, *i*) represent different probability distributions normalized to 1.0, with *i* representing a histogram bin index and *t* representing the time at which the histogram is being constructed (i.e., all data from time 0 to *t*). Histograms were constructed using a Gaussian kernel density estimator to exclude the bins with no population. It has been used in previous studies as a measure of convergence [43,44]. *grcarma* [59,60] was used to generate the highest populated clusters using the top three principal components (PC) and write their representative structures in pdb format files. An important aspect of an aMD calculation is to reweight the distribution to remove the effect of boost applied to the system and to recover the original free energy landscapes. To recover this distribution, ‘amd.log’ is generated during the run that contains information about the extra boost added to each snapshot of the trajectory. Theoretically, aMD simulations can be reweighted by the Boltzmann factors of the corresponding boost potential (i.e., e^Δ*V*/*k*^_B_*T*) and averaged over each bin of selected reaction coordinate(s) to obtain the canonical ensemble, a technique named as exponential average which suffers from large statistical noise especially if higher boost was applied. To overcome this problem, a method named Maclaurin series expansion was used, which approximates the exponential Boltzmann factor and reduces energetic noise considerably [24]. A third algorithm named cumulant expansion (first, second, and third order) was reported to be the most accurate by Miao et al. [61]. While this study reported that cumulant expansion to the second order is most accurate when the boost potential follows near-Gaussian distribution, Jing et al. [62] argued that boost potential should exactly be Gaussian or else this method may show considerable deviation. Maclaurin series expansion up to the 10th order was found to be the most accurate reweighting procedure for all simulations.

## 4. Conclusions

In this study, we have characterized an 18-mer peptaibol named TPN XIIc for its structural and dynamical properties. The peptide shows different folding behavior in water and chloroform solvents. The two largest low-energy clusters obtained from the water system show continuous beta turns, which form a beta-bend ribbon spiral at the *N*-terminal and a 3_10_/α-helical continuous structure at the C-terminal. In chloroform, a higher propensity to form γ-turn at the central Aib12 → Ala10 bond is observed which renders the backbone in a bent state comparable to a beta hairpin. A longer simulation revealed a linear backbone structure that could be attained after crossing a 4 kcal mol^−1^ energy barrier. While addressing the convergence of these two simulations, it was observed that five independent simulations have sampled very similar conformations in water and hence show convergence. While in chloroform, the last 1 µs long simulation samples a very different conformational space not observed previously. The peptide shows a highly dynamic behavior in water compared to chloroform and structural convergence is achieved earlier in the latter. The structural information of these short peptides is crucial to mark functionally important segments. This study is the first step to link the structure to the biological function of tripleurins and to understand their folding mechanisms.

## Figures and Tables

**Table 1:** Average Φ and Ψ angle values for various conformations on the Ramachandran plot (according to Hollingsworth and Karplus) ^13^.

Type	ϕ, ψ Angles
α (alpha helix)	−63, −43
β (beta region)	‒157.2, 161.9
PII-spirals	−65, +145
γ-turns	+80, −80
γ′-turns	−80, +80
δ regions	Extending at 45° angle to the left of α-helix region
δ′ regions	Mirror image of δ region
ζ (pre-proline region)	−130, +80

**Table 2:** Backbone H-bonds of TPN XIIc in explicit water solvent along with their frequency of occurrence given by fraction, average distance, and angle.

Acceptor	Donor	Fraction	Average Distance	Average Angle
Aib1	Div5	0.0560	2.89	162.38
Ser2	Gln6	0.0843	2.88	160.03
Ala3	Gln6	0.1911	2.87	154.95
Ala3	Div7	0.1478	2.88	161.59
Aib4	Div7	0.0296	2.90	155.38
Aib4	Aib8	0.0929	2.89	161.68
Div5	Aib8	0.0242	2.88	154.44
Div5	Val9	0.0271	2.89	161.79
Gln6	Val9	0.1597	2.87	154.92
Gln6	Aib8	0.0545	2.78	149.02
Aib8	Aib12	0.1254	2.89	159.16
Gln6	Ala10	0.1417	2.87	160.11
Div7	Val9	0.0611	2.79	147.89
Div7	Ala10	0.1154	2.88	152.30
Div7	Div11	0.1484	2.89	161.04
Val9	Aib12	0.0456	2.88	159.08
Ala10	Aib12	0.0434	2.80	148.52
Ala10	Leu14	0.0351	2.88	159.89
Div11	Leu14	0.1235	2.86	151.96
Div11	Aib15	0.1890	2.88	162.72
Pro13	Val16	0.1974	2.87	151.15
Pro13	Gln17	0.2110	2.87	159.68
Leu14	Gln17	0.1082	2.88	152.47
Leu14	Pol18	0.4430	2.86	160.43

**Table 3:** Backbone H-bonds of TPN XIIc in explicit chloroform solvent along with their frequency of occurrence given by fraction, average distance, and angle.

Acceptor	Donor	Fraction	Average Distance	Average Angle
Aib1	Div5	0.1538	2.90	162.68
Ser2	Aib4	0.0902	2.82	148.54
Ser2	Gln6	0.2475	2.88	160.23
Ala3	Div5	0.1023	2.80	149.61
Ala3	Div7	0.2394	2.90	162.90
Ala3	Gln6	0.0746	2.88	152.31
Aib4	Aib8	0.0904	2.90	163.41
Gln6	Val9	0.1050	2.89	154.32
Gln6	Div11	0.0767	2.90	152.28
Gln6	Ala10	0.1334	2.87	156.93
Div7	Val9	0.0451	2.91	162.20
Div7	Ala10	0.2448	2.88	152.99
Aib8	Ala10	0.0254	2.88	146.67
Val9	Div11	0.1822	2.83	147.37
Val9	Aib12	0.0186	2.88	161.66
Ala10	Aib12	0.4465	2.79	149.19
Div11	Leu14	0.1384	2.87	155.91
Div11	Aib15	0.0833	2.88	163.24
Aib12	Leu14	0.2099	2.85	151.67
Aib12	Aib15	0.0855	2.89	160.15
Pro13	Aib15	0.1378	2.84	148.97
Pro13	Val16	0.1997	2.88	158.57
Aib15	Pol18	0.2063	2.88	158.80
Leu14	Val16	0.1747	2.81	147.09
Leu14	Gln17	0.2091	2.88	159.58
Leu14	Pol18	0.1474	2.87	161.57
Val16	Pol18	0.0851	2.84	147.39

**Table 4:** Summary of various accelerated molecular dynamics parameters.

Simulations	Time (ns)	Boost Option	Vavg_dihed (kcal mol^−1^)	Vavg_total (kcal mol^−1^)
**In water**	2500 (500 × 3 + 1000 ns)	iamd = 3	210	−25429
**In chloroform**	2500 (500 × 3 + 1000 ns)	iamd = 2	206	−7535

**Table 5:** Summary of coefficient a1, a2 and b1, b2 applied to consecutive simulations and the resulting average boost energy.

Water Simulation			Chloroform Simulation		
a1, a2	b1, b2	Avg. boost (kcal mol^−1^)	a1, a2	b1, b2	Avg. boost (kcal mol^−1^)
4.0	0.16	5	4.0	------	6.5
3.5	0.30	45	4.5	------	10
3.5	0.20	15	6.0	------	30

## Supplementary Materials

The following are available online: which includes detailed explanation for Good-Turing analysis and data of parameterized non-standard amino acid residues, Aib, Div, and Pheol. Figure S1: Potential-of-mean-force values calculated in kcal mol^−1^ as a function of end-to-end distance of TPN XIIc calculated for each step in the trajectory. Figure S2: Estimation of extent of sampling based on Good-Turing formalism (black) water and (red) chloroform.
