# Supplementary material for: Tripleurin XIIc: Peptide Folding Dynamics in Aqueous and Hydrophobic Environment Mimic Using Accelerated Molecular Dynamics
Source: Molecules. 2019 Jan 19;24(2):358. doi: 10.3390/molecules24020358 (PMC6359335; doi:10.3390/molecules24020358)
Supplement: Supplementary file 1 [file molecules-24-00358-s001.pdf]

Tripleurin XIIc: peptide folding dynamics in aqueous and hydrophobic environment mimic using accelerated molecular dynamics

[1] Department of Microbiology, Faculty of Science and Informatics, University of Szeged, Szeged, Közép fasor 52, H-6726 Szeged, Hungary; mariktamas88@gmail.com (T.M.); szandras@bio.u-szeged.hu (A.S.); mucor1959@gmail.com (C.V.); kredics@bio.u-szeged.hu (L.K.)
[2] Doctoral School of Biology, Faculty of Science and Informatics, University of Szeged, Szeged, Közép fasor 52, H-6726 Szeged, Hungary
[3] Institute of Biochemistry, Biological Research Centre, Szeged, Temesvári krt. 62, H-6726 Szeged, Hungary; otvos@brc.hu
* Correspondence: cheta231@gmail.com; Tel.: +36-62-544005

Figure S1: Potential-of-mean-force values calculated in kcal mol^{-1} as a function of end-to-end distance of TPN XIIc calculated for each step in the trajectory. The PMF values were calculated using Maclaurin series expansion method for reweighting end-to-end distance values. All throughout the study it should be noted that backbone curvature is a rather important structural characteristic of TPN XIIc. For water, the energy minima are obtained for structures that are slightly bent, i.e., end-to-end values are from ~18 to 21 Å as shown by two salmon-tinted peptides. For chloroform, the deepest energy minima are obtained for structures showing complete backbone reversal as the two olive-tinted structures, i.e., end-to-end value of 8 Å which denotes unfolded conformation and 14 Å which denotes folded conformation. The third structure with 27 Å is the linear backbone conformation that is obtained after crossing an energy barrier of ~5kcal mol^{-1} and lies at 4 kcal mol^{-1}. Such a rare event was not sampled in previous 500 ns long simulations and occurs only during long 1 μs simulation using aggressive boost parameters.

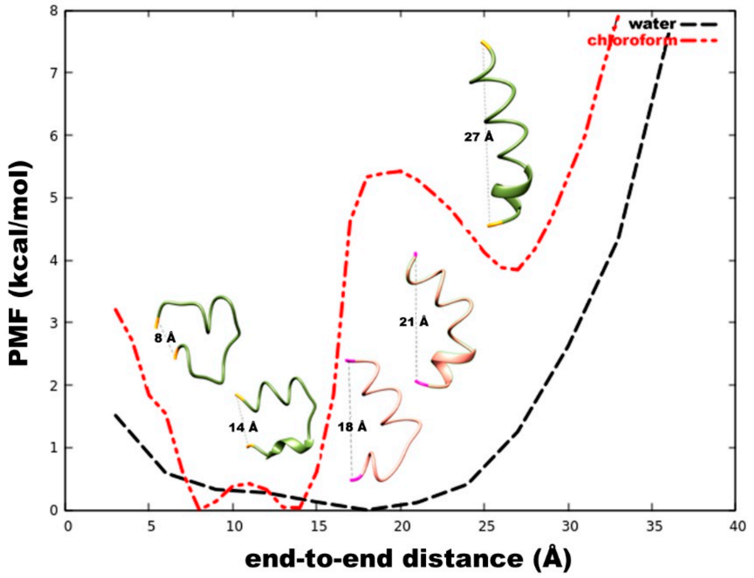

Figure S1: Potential-of-mean-force values calculated in kcal mol−1 as a function of end-to-end distance of TPN XIIc calculated for each step in the trajectory.

Good-Turing analysis

To address the question of convergence, a relatively new technique based on probability theory reported by Koukos et al., 2012 [1] was applied to all simulations. They (in the manuscript) argued that any meaningful

way to deduce convergence must be based on probability theory. They devised Good-Turing formalism for estimation of frequency of unobserved species (of conformations) in a trajectory. This formalism draws its conclusion on the classical RMSD (root-mean-square-deviation) matrix by answering the following question "What is the probability that a molecular configuration with an RMSD (from all other already observed configurations) higher than a given threshold has not actually been observed?". The algorithm can be accessed as an R script and applied using 'grcarma' on trajectories of any length. In simple terms, this algorithm starts by quantifying structural distance in form of RMSD (root-mean-square deviation) by creating an RMSD matrix (original matrix [N x N]) from the trajectory. This maximum RMSD is the highest RMSD observed between successive structures in the original matrix i.e. sampling factor of 1. A sub-matrix formed of size [N/2 x N/2] from the original matrix by taking every second row and column and maximum RMSDs determined on its superdiagonal means that sampling factor of 2 was applied. For real time application, the trajectories should be spaced in a way that the sampling factor stays close to 1. The resulting matrix is considered as a distance matrix and a dendrogram is constructed using hierarchical clustering methods[47]. This dendrogram is used to produce clusters at different RMSD cutoffs along with their frequencies. Finally, the Good-Turing formalism is applied to these frequencies to calculate the probability of unobserved species (P_unobs.) that could be observed if the simulations were extended. In simple terms, this analysis indicates the probability of observing a molecular configuration not observed before [2, 3]

In case of water simulation (Figure S2), the most distinct structure we can expect to observe if we double the simulation time will differ by no more than approximately 3.9 Å (RMSD) from those already observed. To look at it another way, one out of every five (probability of unobserved species, P_unobs = 0.20) new structures encountered will differ by an RMSD of at least 3 Å. On the other hand, simulation in chloroform indicated towards slightly higher convergence as the maximum RMSD value of an unobserved species was calculated to be only 3.07 Å, i.e. one in eleven structures (P_unobs = 0.11) shall differ only by 2.8 Å from the conformations already observed. Keep in mind that this calculation includes all possible unfolded conformations and the high RMSD probably indicates towards different unfolded structures that have not been encountered. But such unfolded states are not required when we are studying the folding dynamics of a peptide. This method is an assessment of structural convergence of peptide dynamics based on the statistics obtained from previously sampled conformations. It is a straightforward method using RMSD as the base value which is an easy but not always accurate measure of structural similarity. Nevertheless, such an analysis along with convergence of the principal component space indicates that all major metastable states of TPN XIIc peptide in water and chloroform have been sampled using accelerated MD. The probability of unobserved species reaches 0 at the maximum RMSD value that could be observed if the simulation is extended. Evidently, this value seems to be higher in water than in chloroform.

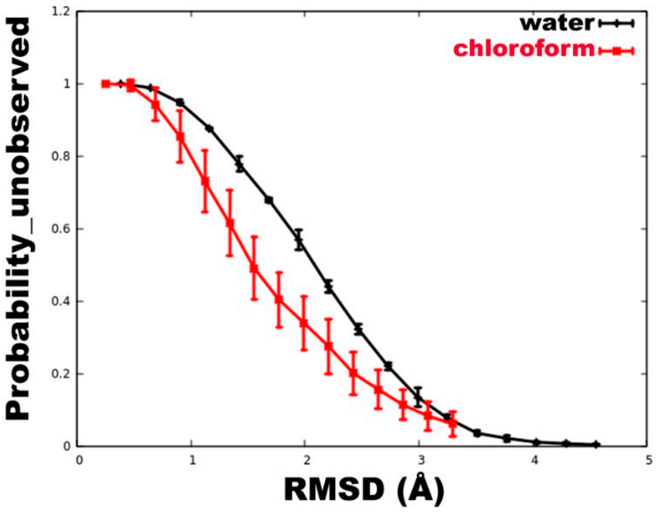

Figure S2: Estimation of extent of sampling based on Good-Turing formalism (black) water and (red) chloroform.

1. Koukos, P. I.; Glykos, N. M., On the application of Good-Turing statistics to quantify convergence of biomolecular simulations. *J Chem Inf Model* **2014,** 54, (1), 209-17.
2. Serafeim, A. P.; Salamanos, G.; Patapati, K. K.; Glykos, N. M., Sensitivity of Folding Molecular Dynamics Simulations to Even Minor Force Field Changes. *J Chem Inf Model* **2016,** 56, (10), 2035-2041.
3. Shao, J.; Tanner, S. W.; Thompson, N.; Cheatham, T. E., Clustering Molecular Dynamics Trajectories: 1. Characterizing the Performance of Different Clustering Algorithms. *J Chem Theory Comput* **2007,** 3, (6), 2312-34.

Detailed description for residue parameterization

The non-standard residues, Aib (aminoisobutyric acid) and Div (D-isovaline) were parameterized as part of one calculation along with 4 standard residues, alanine, serine, glycine and valine. The parameter file, .frcmod, is calculated collectively for all these residues and is provided below. To create their amber libraries in .off format, the respective structures of these residues were loaded using a leap script provided by R.E.D server. The reason for collective parameterization was to be able to compare the charges for standard residues with preexisting libraries of AmberTools16.

Note: The mol2 files contain information of Aib and Div as residue units with free ends that can be readily used in building a peptide sequence. They were parameterized as ACE-AIB/DIV-NME units.

Using leap,

loadAmberParams frcmod.known #parameter file for all 6 residues
AIB = loadmol3 m3-c1_f2.mol2 #from R.E.D charge calculation results
DIV = loadmol3 m6-c1_f2.mol2

saveoff AIB AIB.off #saving amber libraries
saveoff DIV DIV.off

The .mol2 files, for AIB

```
@<TRIPOS>MOLECULE
F08
    13    12     1     0     1
SMALL
USER_CHARGES
@<TRIPOS>ATOM
   1 N1     1.179404   0.787225  -0.289608 N    1   F08 -0.4552  0.0000 ****
   2 H7     1.401639   1.218298  -1.161329 H    1   F08  0.2870  0.0000 ****
   3 C1    -0.246026   0.712307  -0.009072 CT   1   F08  0.1590  0.0000 ****
   4 C2    -0.513722   0.943419   1.490893 CT   1   F08 -0.1445  0.0000 ****
   5 C3    -0.874452   1.859278  -0.822371 CT   1   F08 -0.1445  0.0000 ****
   6 H8    -0.683793   1.725172  -1.881967 HC   1   F08  0.0464  0.0000 ****
   7 H9    -1.944751   1.917349  -0.681560 HC   1   F08  0.0464  0.0000 ****
   8 H10   -0.438130   2.800958  -0.509348 HC   1   F08  0.0464  0.0000 ****
   9 C6    -0.815324  -0.626254  -0.549026 C    1   F08  0.5633  0.0000 ****
  10 O     -0.117607  -1.349894  -1.206062 O    1   F08 -0.5435  0.0000 ****
  11 H1    -1.529198   1.266287   1.670775 HC   1   F08  0.0464  0.0000 ****
  12 H2     0.148540   1.726800   1.836879 HC   1   F08  0.0464  0.0000 ****
  13 H     -0.336510   0.060129   2.090571 HC   1   F08  0.0464  0.0000 ****
@<TRIPOS>BOND
    1     1     2 1
    2     1     3 1
    3     3     4 1
    4     3     5 1
    5     3     9 1
    6     4    11 1
    7     4    12 1
    8     4    13 1
    9     5     6 1
   10     5     7 1
   11     5     8 1
   12     9    10 1
@<TRIPOS>SUBSTRUCTURE
      1 F08              1 ****               0 ****  ****
@<TRIPOS>HEADTAIL
N1 1
C6 1
@<TRIPOS>RESIDUECONNECT
1 N1 C6 0 0 0 0
```

D-isovaline (DIV)

```
@<TRIPOS>MOLECULE
F17
```

```
    16    15     1     0     1
SMALL
USER_CHARGES
@<TRIPOS>ATOM
   1 N1      1.069412   0.434947   0.415318 N     1    F17 -0.3647  0.0000 ****
   2 H8      0.918993   1.367037   0.740563 H     1    F17  0.2358  0.0000 ****
   3 C1     -0.156337  -0.267531   0.058905 CT    1    F17  0.0552  0.0000 ****
   4 C2     -0.255076  -1.597290   0.828207 CT    1    F17 -0.2071  0.0000 ****
   5 C3     -0.280048  -0.476971  -1.472639 CT    1    F17 -0.0427  0.0000 ****
   6 C4     -0.175361   0.801565  -2.304979 CT    1    F17 -0.0917  0.0000 ****
   7 H9     -0.971825   1.500344  -2.073312 HC    1    F17  0.0282  0.0000 ****
   8 H10    -0.246482   0.559673  -3.360441 HC    1    F17  0.0282  0.0000 ****
   9 H11     0.768238   1.306343  -2.139664 HC    1    F17  0.0282  0.0000 ****
  10 C7     -1.286111   0.698418   0.513586 C     1    F17  0.6153  0.0000 ****
  11 O      -1.017880   1.799366   0.916720 O     1    F17 -0.5641  0.0000 ****
  12 H1      0.491864  -1.170168  -1.784452 HC    1    F17  0.0407  0.0000 ****
  13 H2     -1.219741  -0.969252  -1.691480 HC    1    F17  0.0407  0.0000 ****
  14 H3     -1.133983  -2.164910   0.562186 HC    1    F17  0.0660  0.0000 ****
  15 H4     -0.269747  -1.411989   1.896661 HC    1    F17  0.0660  0.0000 ****
  16 H       0.598634  -2.222187   0.612382 HC    1    F17  0.0660  0.0000 ****
@<TRIPOS>BOND
    1     1     2 1
    2     1     3 1
    3     3     4 1
    4     3     5 1
    5     3    10 1
    6     4    14 1
    7     4    15 1
    8     4    16 1
    9     5     6 1
   10     5    12 1
   11     5    13 1
   12     6     7 1
   13     6     8 1
   14     6     9 1
   15    10    11 1
@<TRIPOS>SUBSTRUCTURE
      1 F17              1 ****               0 ****  ****
@<TRIPOS>HEADTAIL
N1 1
C7 1
@<TRIPOS>RESIDUECONNECT
1 N1 C7 0 0 0 0
```

The parameter file used for both residues

```
FRCMOD file generated by PyRED version SEP-2015 - q4md-forcefieldtools.org
MASS      mass              pol            Source
C         12.010            0.616          taken from parm10.dat
CT        12.010            0.878          taken from parm10.dat
CX        12.010            0.360          taken from parm10.dat
H          1.008            0.161          taken from parm10.dat
H1         1.008            0.135          taken from parm10.dat
HC         1.008            0.135          taken from parm10.dat
HO         1.008            0.135          taken from parm10.dat
N         14.010            0.530          taken from parm10.dat
O         16.000            0.434          taken from parm10.dat
OH        16.000            0.465          taken from parm10.dat

BOND   K(kcal.mol-1.ang-2)  Dist0(ang)     Source
C -CT     315.0             1.522          adapted from parm10.dat 317.0
C -CX     315.0             1.522          adapted from parm10.dat 317.0
C -N      490.0             1.335          taken from parm10.dat
```

```
C -O      570.0              1.229          taken from parm10.dat
CT-CT     310.0              1.526          taken from parm10.dat
CT-CX     310.0              1.526          taken from parm10.dat
CT-H1     340.0              1.090          taken from parm10.dat
CT-HC     340.0              1.090          taken from parm10.dat
CT-N      335.0              1.449          adapted from parm10.dat 337.0
CT-OH     320.0              1.410          taken from parm10.dat
CX-H1     340.0              1.090          taken from parm10.dat
CX-N      335.0              1.449          adapted from parm10.dat 337.0
H -N      435.0              1.010          adapted from parm10.dat 434.0
HO-OH     555.0              0.960          adapted from parm10.dat 553.0

ANGLE  K(kcal.mol-1.rad-2)  Theta0(deg)    Source
CT-C -N        70.0          116.60         taken from parm10.dat
CT-C -O        80.0          120.40         taken from parm10.dat
CX-C -N        70.0          116.60         taken from parm10.dat
CX-C -O        80.0          120.40         taken from parm10.dat
N -C -O        80.0          122.90         taken from parm10.dat
C -CT-CT       65.0          111.10         adapted from parm10.dat 63.0
C -CT-HC       50.0          109.50         taken from parm10.dat
C -CT-N        65.0          110.10         adapted from parm10.dat 63.0
CT-CT-CT       40.0          109.50         taken from parm10.dat
CT-CT-CX       40.0          109.50         taken from parm10.dat
CT-CT-HC       50.0          109.50         taken from parm10.dat
CT-CT-N        80.0          109.70         taken from parm10.dat
CX-CT-H1       50.0          109.50         taken from parm10.dat
CX-CT-HC       50.0          109.50         taken from parm10.dat
CX-CT-OH       50.0          109.50         taken from parm10.dat
H1-CT-H1       35.0          109.50         taken from parm10.dat
H1-CT-N        50.0          109.50         taken from parm10.dat
H1-CT-OH       50.0          109.50         taken from parm10.dat
HC-CT-HC       35.0          109.50         taken from parm10.dat
C -CX-CT       65.0          111.10         adapted from parm10.dat 63.0
C -CX-H1       50.0          109.50         taken from parm10.dat
C -CX-N        65.0          110.10         adapted from parm10.dat 63.0
CT-CX-H1       50.0          109.50         taken from parm10.dat
CT-CX-N        80.0          109.70         taken from parm10.dat
H1-CX-H1       35.0          109.50         taken from parm10.dat
H1-CX-N        50.0          109.50         taken from parm10.dat
C -N -CT       50.0          121.90         taken from parm10.dat
C -N -CX       50.0          121.90         taken from parm10.dat
C -N -H        50.0          120.00         taken from parm10.dat
CT-N -H        50.0          118.04         taken from parm10.dat
CX-N -H        50.0          118.04         taken from parm10.dat
CT-OH-HO       55.0          108.50         taken from parm10.dat

DIHEDRAL    Path V(kcal.mol-1.rad-1) Phase(deg.) Period Source
N -C -CT-CT   1    0.00000000e+00        0.0       -4.   taken from parm10.dat
N -C -CT-CT   1    4.00000000e-01        0.0       -3.   taken from parm10.dat
N -C -CT-CT   1    2.00000000e-01        0.0       -2.   taken from parm10.dat
N -C -CT-CT   1    2.00000000e-01        0.0        1.   taken from parm10.dat
N -C -CT-HC   1    0.00000000e+00        0.0        2.   adapted from parm10.dat
i.e X-C-CT-X   0.0/6
N -C -CT-N    1    0.00000000e+00        0.0        2.   adapted from parm10.dat
i.e X-C-CT-X   0.0/6
O -C -CT-CT   1    0.00000000e+00        0.0        2.   adapted from parm10.dat
i.e X-C-CT-X   0.0/6
O -C -CT-HC   1    8.00000000e-01        0.0       -1.   taken from parm10.dat
O -C -CT-HC   1    0.00000000e+00        0.0       -2.   taken from parm10.dat
O -C -CT-HC   1    8.00000000e-02      180.0        3.   taken from parm10.dat
O -C -CT-N    1    0.00000000e+00        0.0        2.   adapted from parm10.dat
i.e X-C-CT-X   0.0/6
N -C -CX-CT   1    0.00000000e+00        0.0       -4.   taken from parm10.dat
N -C -CX-CT   1    4.00000000e-01        0.0       -3.   taken from parm10.dat
```

```
N -C -CX-CT   1    2.00000000e-01         0.0       -2.   taken from parm10.dat
N -C -CX-CT   1    2.00000000e-01         0.0        1.   taken from parm10.dat
N -C -CX-H1   1    0.00000000e+00         0.0        2.   adapted from parm10.dat
i.e X-C-CX-X   0.0/6
N -C -CX-N    1    0.00000000e+00         0.0       -4.   taken from parm10.dat
N -C -CX-N    1    5.50000000e-01       180.0       -3.   taken from parm10.dat
N -C -CX-N    1    1.58000000e+00       180.0       -2.   taken from parm10.dat
N -C -CX-N    1    4.50000000e-01       180.0        1.   taken from parm10.dat
O -C -CX-CT   1    0.00000000e+00         0.0        2.   adapted from parm10.dat
i.e X-C-CX-X   0.0/6
O -C -CX-H1   1    8.00000000e-01         0.0       -1.   taken from parm10.dat
O -C -CX-H1   1    0.00000000e+00         0.0       -2.   taken from parm10.dat
O -C -CX-H1   1    8.00000000e-02       180.0        3.   taken from parm10.dat
O -C -CX-N    1    0.00000000e+00         0.0        2.   adapted from parm10.dat
i.e X-C-CX-X   0.0/6
CT-C -N -CT   1    2.50000000e+00       180.0        2.   adapted from parm10.dat
i.e X-C-N-X   10.0/4
CT-C -N -CX   1    2.50000000e+00       180.0        2.   adapted from parm10.dat
i.e X-C-N-X   10.0/4
CT-C -N -H    1    2.50000000e+00       180.0        2.   adapted from parm10.dat
i.e X-C-N-X   10.0/4
CX-C -N -CT   1    2.50000000e+00       180.0        2.   adapted from parm10.dat
i.e X-C-N-X   10.0/4
CX-C -N -H    1    2.50000000e+00       180.0        2.   adapted from parm10.dat
i.e X-C-N-X   10.0/4
O -C -N -CT   1    2.50000000e+00       180.0        2.   adapted from parm10.dat
i.e X-C-N-X   10.0/4
O -C -N -CX   1    2.50000000e+00       180.0        2.   adapted from parm10.dat
i.e X-C-N-X   10.0/4
O -C -N -H    1    2.50000000e+00       180.0       -2.   taken from parm10.dat
O -C -N -H    1    2.00000000e+00         0.0        1.   taken from parm10.dat
C -CT-CT-CT   1    1.55555556e-01         0.0        3.   adapted from parm10.dat
i.e X-CT-CT-X   1.4/9
C -CT-CT-HC   1    1.55555556e-01         0.0        3.   adapted from parm10.dat
i.e X-CT-CT-X   1.4/9
CT-CT-CT-CT   1    1.80000000e-01         0.0       -3.   taken from parm10.dat
CT-CT-CT-CT   1    2.50000000e-01       180.0       -2.   taken from parm10.dat
CT-CT-CT-CT   1    2.00000000e-01       180.0        1.   taken from parm10.dat
CT-CT-CT-HC   1    1.60000000e-01         0.0        3.   taken from parm10.dat
CT-CT-CT-N    1    1.55555556e-01         0.0        3.   adapted from parm10.dat
i.e X-CT-CT-X   1.4/9
CX-CT-CT-HC   1    1.60000000e-01         0.0        3.   taken from parm10.dat
HC-CT-CT-HC   1    1.50000000e-01         0.0        3.   taken from parm10.dat
HC-CT-CT-N    1    1.55555556e-01         0.0        3.   adapted from parm10.dat
i.e X-CT-CT-X   1.4/9
CT-CT-CX-C    1    1.55555556e-01         0.0        3.   adapted from parm10.dat
i.e X-CT-CX-X   1.4/9
CT-CT-CX-H1   1    1.55555556e-01         0.0        3.   adapted from parm10.dat
i.e X-CT-CX-X   1.4/9
CT-CT-CX-N    1    1.55555556e-01         0.0        3.   adapted from parm10.dat
i.e X-CT-CX-X   1.4/9
H1-CT-CX-C    1    1.55555556e-01         0.0        3.   adapted from parm10.dat
i.e X-CT-CX-X   1.4/9
H1-CT-CX-H1   1    1.55555556e-01         0.0        3.   adapted from parm10.dat
i.e X-CT-CX-X   1.4/9
H1-CT-CX-N    1    1.55555556e-01         0.0        3.   adapted from parm10.dat
i.e X-CT-CX-X   1.4/9
HC-CT-CX-C    1    1.55555556e-01         0.0        3.   adapted from parm10.dat
i.e X-CT-CX-X   1.4/9
HC-CT-CX-H1   1    1.55555556e-01         0.0        3.   adapted from parm10.dat
i.e X-CT-CX-X   1.4/9
HC-CT-CX-N    1    1.55555556e-01         0.0        3.   adapted from parm10.dat
i.e X-CT-CX-X   1.4/9
OH-CT-CX-C    1    1.55555556e-01         0.0        3.   adapted from parm10.dat
```

```
i.e X-CT-CX-X   1.4/9
OH-CT-CX-H1   1    0.00000000e+00        0.0       -3.   taken from parm10.dat
OH-CT-CX-H1   1    2.50000000e-01        0.0        1.   taken from parm10.dat
OH-CT-CX-N    1    1.55555556e-01        0.0        3.   adapted from parm10.dat
i.e X-CT-CX-X   1.4/9
C -CT-N -C    1    0.00000000e+00        0.0        2.   adapted from parm10.dat
i.e X-CT-N-X   0.0/6
C -CT-N -H    1    0.00000000e+00        0.0        2.   adapted from parm10.dat
i.e X-CT-N-X   0.0/6
CT-CT-N -C    1    0.00000000e+00        0.0       -4.   taken from parm10.dat
CT-CT-N -C    1    4.00000000e-01        0.0       -3.   taken from parm10.dat
CT-CT-N -C    1    2.00000000e+00        0.0       -2.   taken from parm10.dat
CT-CT-N -C    1    2.00000000e+00        0.0        1.   taken from parm10.dat
CT-CT-N -H    1    0.00000000e+00        0.0        2.   adapted from parm10.dat
i.e X-CT-N-X   0.0/6
H1-CT-N -C    1    0.00000000e+00        0.0        2.   adapted from parm10.dat
i.e X-CT-N-X   0.0/6
H1-CT-N -H    1    0.00000000e+00        0.0        2.   adapted from parm10.dat
i.e X-CT-N-X   0.0/6
CX-CT-OH-HO   1    1.60000000e-01        0.0       -3.   taken from parm10.dat
CX-CT-OH-HO   1    2.50000000e-01        0.0        1.   taken from parm10.dat
H1-CT-OH-HO   1    1.66666667e-01        0.0        3.   adapted from parm10.dat
i.e X-CT-OH-X   0.5/3
C -CX-N -C    1    0.00000000e+00        0.0       -4.   taken from parm10.dat
C -CX-N -C    1    4.20000000e-01        0.0       -3.   taken from parm10.dat
C -CX-N -C    1    2.70000000e-01        0.0       -2.   taken from parm10.dat
C -CX-N -C    1    0.00000000e+00        0.0        1.   taken from parm10.dat
C -CX-N -H    1    0.00000000e+00        0.0        2.   adapted from parm10.dat
i.e X-CX-N-X   0.0/6
CT-CX-N -C    1    0.00000000e+00        0.0       -4.   taken from parm10.dat
CT-CX-N -C    1    4.00000000e-01        0.0       -3.   taken from parm10.dat
CT-CX-N -C    1    2.00000000e+00        0.0       -2.   taken from parm10.dat
CT-CX-N -C    1    2.00000000e+00        0.0        1.   taken from parm10.dat
CT-CX-N -H    1    0.00000000e+00        0.0        2.   adapted from parm10.dat
i.e X-CX-N-X   0.0/6
H1-CX-N -C    1    0.00000000e+00        0.0        2.   adapted from parm10.dat
i.e X-CX-N-X   0.0/6
H1-CX-N -H    1    0.00000000e+00        0.0        2.   adapted from parm10.dat
i.e X-CX-N-X   0.0/6

IMPROPER         V(kcal.mol-1.rad-1) Phase(deg.) Period Source
CT-N -C -O         1.05000000e+01      180.0       2.   adapted from parm10.dat
i.e X-X-C-O
CX-N -C -O         1.05000000e+01      180.0       2.   adapted from parm10.dat
i.e X-X-C-O
C -CT-N -H         1.10000000e+00      180.0       2.   taken from parm10.dat
C -CX-N -H         1.10000000e+00      180.0       2.   taken from parm10.dat

NONBON      R*(ang)   Eps(kcal.mol-1)       Source
C           1.9080    0.08600000            taken from parm10.dat
CT          1.9080    0.10940000            taken from parm10.dat
CX          1.9080    0.10940000            taken from parm10.dat
H           0.6000    0.01570000            taken from parm10.dat
H1          1.3870    0.01570000            taken from parm10.dat
HC          1.4870    0.01570000            taken from parm10.dat
HO          0.0000    0.00000000            taken from parm10.dat
N           1.8240    0.17000000            taken from parm10.dat
O           1.6612    0.21000000            taken from parm10.dat
OH          1.7210    0.21040000            taken from parm10.dat
```

Pol/Pheol (Phenylalaninol) parameter file

```
MASS
N  14.010        0.530               same as n
H  1.008         0.161               same as hn
CT 12.010        0.878               same as c3
H1 1.008         0.135               same as hc
HC 1.008         0.135               same as hc
CA 12.010        0.360               same as c2
HA 1.008         0.135               same as hc
OH 16.000        0.465               same as oh
HO 1.008         0.135               same as ho

BOND
N -H   410.20   1.009       same as hn-n
N -CT  330.60   1.460       same as c3-n
CT-H1  337.30   1.092       same as c3-hc
CT-CT  303.10   1.535       same as c3-c3
CT-HC  337.30   1.092       same as c3-hc
CT-CA  328.30   1.508       same as c2-c3
CA-CA  478.40   1.387       same as ca-ca
CA-HA  344.30   1.087       same as c2-hc
CT-OH  314.10   1.426       same as c3-oh
OH-HO  369.60   0.974       same as ho-oh

ANGLE
N -CT-H1   49.800     109.500   same as hc-c3-n
N -CT-CT   65.900     112.130   same as c3-c3-n
H -N -CT   46.000     116.780   same as c3-n -hn
CT-CT-HC   46.400     110.050   same as c3-c3-hc
CT-CT-CA   63.700     110.960   same as c2-c3-c3
CT-CT-H1   46.400     110.050   same as c3-c3-hc
CT-CT-OH   67.700     109.430   same as c3-c3-oh
CT-CT-CT   63.200     110.630   same as c3-c3-c3
CT-CA-CA   64.300     123.420   same as c2-c2-c3
HC-CT-HC   39.400     108.350   same as hc-c3-hc
HC-CT-CA   47.000     110.490   same as c2-c3-hc
CA-CA-HA   50.300     119.700   same as c2-c2-hc
CA-CA-CA   67.200     119.970   same as ca-ca-ca
CT-OH-HO   47.100     108.160   same as c3-oh-ho
H1-CT-H1   39.400     108.350   same as hc-c3-hc
H1-CT-OH   51.100     109.500   same as hc-c3-oh

DIHEDRAL
N -CT-CT-HC   1    0.156         0.000          3.000      same as X -c3-c3-X
N -CT-CT-CA   1    0.156         0.000          3.000      same as X -c3-c3-X
N -CT-CT-H1   1    0.156         0.000          3.000      same as X -c3-c3-X
N -CT-CT-OH   1    0.156         0.000          3.000      same as X -c3-c3-X
H -N -CT-H1   1    0.000         0.000          2.000      same as X -c3-n -X
H -N -CT-CT   1    0.000         0.000          2.000      same as X -c3-n -X
CT-CT-CA-CA   1    0.000         0.000          2.000      same as X -c2-c3-X
CT-CT-OH-HO   1    0.167         0.000          3.000      same as X -c3-oh-X
H1-CT-CT-HC   1    0.156         0.000          3.000      same as X -c3-c3-X
H1-CT-CT-CA   1    0.156         0.000          3.000      same as X -c3-c3-X
H1-CT-CT-H1   1    0.156         0.000          3.000      same as X -c3-c3-X
H1-CT-CT-OH   1    0.156         0.000          3.000      same as X -c3-c3-X
CT-CT-CT-H1   1    0.156         0.000          3.000      same as X -c3-c3-X
CT-CT-CT-OH   1    0.156         0.000          3.000      same as X -c3-c3-X
CT-CA-CA-HA   1    6.650       180.000          2.000      same as X -c2-c2-X
CT-CA-CA-CA   1    6.650       180.000          2.000      same as X -c2-c2-X
HC-CT-CT-CT   1    0.156         0.000          3.000      same as X -c3-c3-X
HC-CT-CA-CA   1    0.000         0.000          2.000      same as X -c2-c3-X
CA-CT-CT-CT   1    0.156         0.000          3.000      same as X -c3-c3-X
CA-CA-CA-HA   1    6.650       180.000          2.000      same as X -c2-c2-X
CA-CA-CA-CA   1    3.625       180.000          2.000      same as X -ca-ca-X
```

```
HA-CA-CA-HA   1    6.650       180.000           2.000      same as X -c2-c2-X
H1-CT-OH-HO   1    0.167         0.000           3.000      same as X -c3-oh-X

IMPROPER
CA-CA-CA-CT          1.1          180.0         2.0
CA-CA-CA-HA          1.1          180.0         2.0

NONBON
  N           1.8240  0.1700
  H           0.6000  0.0157
  CT          1.9080  0.1094
  H1          1.4870  0.0157
  HC          1.4870  0.0157
  CA          1.9080  0.0860
  HA          1.4870  0.0157
  OH          1.7210  0.2104
  HO          0.0000  0.0000
```
